# Decoupling blood telomere length from age in recipients of allogeneic hematopoietic cell transplant in the BMT-CTN 1202

**DOI:** 10.3389/fimmu.2022.966301

**Published:** 2022-10-03

**Authors:** Tsung-Po Lai, Simon Verhulst, Casey L. Dagnall, Amy Hutchinson, Stephen R. Spellman, Alan Howard, Hormuzd A. Katki, John E. Levine, Wael Saber, Abraham Aviv, Shahinaz M. Gadalla

**Affiliations:** ^1^ Center of Human Development and Aging, New Jersey Medical School, Rutgers, The State University of New Jersey, Newark, NJ, United States; ^2^ Groningen Institute for Evolutionary Life Sciences, University of Groningen, Groningen, Netherlands; ^3^ Division of Cancer Epidemiology and Genetics, National Cancer Institute, Bethesda, MD, United States; ^4^ Leidos Biomedical Research, Inc., Frederick National Laboratory for Cancer Research, Frederick, MD, United States; ^5^ Center for International Blood and Marrow Transplant Research, National Marrow Donor Program, Minneapolis, MN, United States; ^6^ Tisch Cancer Institute, Icahn School of Medicine at Mount Sinai, New York, NY, United States; ^7^ Center for International Blood and Marrow Transplant Research, Medical College of Wisconsin, Milwaukee, WI, United States

**Keywords:** donor selection, allogeneic hematopoietic cell transplant, telomere length (TL), telomere length assay, aging, telomere length dynamics

## Abstract

The age of allogeneic hematopoietic cell transplant (HCT) donors and their hematopoietic cell telomere length (TL) might affect recipients’ outcomes. Our goals were to examine the possible effect of these donors’ factors on the recipients’ hematopoietic cell TL and quantify hematopoietic cell TL shortening in the critical first three-month post-HCT. We measured hematopoietic cell TL parameters in 75 recipient-donor pairs, from the Blood and Marrow Transplant Clinical Trials Network (protocol#1202), by Southern blotting (SB), the Telomeres Shortest Length Assay (TeSLA), and quantitative PCR (qPCR). Recipients’ hematopoietic cell TL parameters post-HCT correlated with donors’ age (p<0.001 for all methods), but not recipients’ own age, and with donors’ pre-HCT hematopoietic cell TL (p<0.0001 for all). Multivariate analyses showed that donors’ hematopoietic cell TL pre-HCT, independent of donors’ age, explained most of the variability in recipients’ hematopoietic cell TL post-HCT (81% for SB, 56% for TeSLA, and 65% for qPCR; p>0.0001 for all). SB and TeSLA detected hematopoietic cell TL shortening in all recipients post-HCT (mean=0.52kb and 0.47kb, respectively; >15-fold the annual TL shortening in adults; p<0.00001 for both), but qPCR detected shortening only in 57.5% of recipients. TeSLA detected a buildup of post-HCT of telomeres <3 kb in 96% of recipients (p<0.0001). In conclusion, HCT decouples hematopoietic cell TL in the recipients from their own age to reflect the donors’ age. The potential donors’ age effect on outcomes of HCT might be partially mediated by short hematopoietic cell TL in older donors. qPCR-based TL measurement is suboptimal for detecting telomere shortening post-HCT.

## Introduction

Survival of allogeneic hematopoietic cell transplant (HCT) recipients has progressively improved over the years, mainly because of the judicious choice of donors based on high resolution human leukocyte antigen (HLA) typing, and the use of less toxic regimens, selective immunosuppressive therapies, and better infection prophylaxis (1). However, allogeneic HCT recipients still suffer from high mortality risk post-transplant (2). Older donors’ age was shown to contribute to this risk, although the underlying mechanisms are not well understood (3, 4). We propose that recipients of HCT from older adults are likely to have shorter hematopoietic cell telomeres than recipients of HCT from younger adults, explaining in part less favorable outcome in the former group.

The possible role of donors’ hematopoietic cell telomere length (TL) in recipients’ outcomes after allogeneic HCT has been investigated in several studies. Patients with severe aplastic anemia (SAA) who received hematopoietic cells with longer telomeres (measured by the high throughput qPCR assay) from unrelated donors displayed better survival (hazard ratio, HR=0.61, p=0.006, for the top tertile vs. the bottom two tertiles of donor hematopoietic cell TL) (5). A follow-up study in a subset of these HCT recipients showed a 20% mortality risk reduction for every kb longer donors’ hematopoietic cell TL, measured by the clinically-used flow fluorescent *in-situ* hybridization (flow FISH) method (6). In yet another study, the five-year survival significantly improved from 65% to 95% in SAA patients receiving HCT from matched sibling whose hematopoietic cell TL, measured by qPCR, was ranked in the longest quartile (7). No five-year mortality risk reduction was observed in patients with acute leukemia who received HCT from donors with long hematopoietic cell telomeres, measured by qPCR (8). The observed TL-related survival discrepancy between HCT recipients for SAA vs. acute leukemia may stem in part from the underlying malignancy driving post-HCT mortality, differences in post-transplant hematopoietic cell TL dynamics, and/or sub-optimal TL measurement methods. These findings underscore the need to identify optimal TL measurement methods that will generate further insight into hematopoietic cell TL dynamics post-HCT. Some of these methods might be ultimately useful in selecting donors for HCT.

Post-transplant hematopoietic cell TL dynamics were examined, using Southern blotting (SB) or Flow FISH, in several small studies (comprising 11-36 recipients), performed two or more decades ago. Results indicate that the donors’ hematopoietic cell TL shortened in the recipients at a magnitude of 0.3-2 kilobase (kb) within the first two years after HCT (9–13). A more recent study of 13 recipients of double-unit cord blood transplant showed an average of 3 kb hematopoietic cell TL shortening within a year after transplant (14). These magnitudes of hematopoietic cell TL shortening are up to hundredfold higher than the ~ 0.03 kb yearly shortening of hematopoietic cell TL during adulthood (15). In addition, a recent study showed an increased mortality risk in HCT recipients for hematologic malignancies, whose hematopoietic cell TL, measured by qPCR, experienced faster shortening during a 9–15 month period post HCT (16). The TL dynamics early post-HCT, or factors affecting its observed changes are still unclear.

None of the studies above examined hematopoietic cell TL dynamics during the first three months post-HCT. During this critical period, the donors’ hematopoietic cells massively proliferate to regenerate the recipients’ hematopoietic system (17), and thus might experience a substantial telomere shortening. Therefore, our first goal was to examine the extent that the donors’ hematopoietic cell TL and age define hematopoietic cell TL in the recipients after the first three months post-HCT. Our second goal was to examine which TL measurement method is optimal for producing reliable sequential TL data that capture this shortening. This is particularly relevant, since qPCR-based methodology has become the principal way of measuring TL in population-based research, and considering the recent development of the Telomeres Shortest Length Assay (TeSLA) (18, 19). We thus measured TL parameters by three methods: Southern blotting of the terminal restriction fragments (SB; the ‘gold standard’ method) (20), qPCR, and TeSLA.

## Methods

### HCT recipients and donors

Our study includes 75 consecutive recipient-donor pairs of allogeneic HCT. Of these 45 were unrelated pairs and 30 were related pairs (25 sibling and 5 haploidentical donors). Transplants were performed between December 2013 to March 2014. All recipients reached neutrophil engraftment and had a blood sample collected at three months post-HCT as part of the Blood and Marrow Transplant Clinical Trial Network protocol#1202 (https://clinicaltrials.gov/ct2/show/NCT01879072). Donors’ samples pre-HCT were provided by the Center for International Blood and Marrow Transplant Research. (https://www.cibmtr.org/Samples/Pages/index.aspx). Donors’ information and pre-HCT blood samples were provided by the Center for International Blood and Marrow Transplant Research (https://www.cibmtr.org/Samples/Pages/index.aspx).

### Telomere length measurements

DNA was extracted from previously collected peripheral blood samples (frozen whole blood or buffy coat) using the Mag-Bind Blood & Tissue Kit (Omega Bio-Tek) on the KingFisher Flex (ThermoFisher Scientific) instrument. Donors’ samples were collected within one month before bone marrow aspiration for bone marrow grafts, or stem cell mobilization for peripheral blood stem cell grafts. All DNA samples passed an integrity test using a 1% (w/v) agarose gel.

SB was performed as previously described (20). Briefly, DNA was digested using the Hinf I and Rsa I restriction enzymes (Roche Applied Sciences, Mannheim, Germany). The terminal restriction fragments and DNA ladders were resolved on 0.5% agarose gels for 16 h (2 V/cm). Each sample was measured in duplicate.

TeSLA measurements were performed as previously described (19). Briefly, extracted DNA was ligated at the overhangs of telomeres to single-stranded adaptors. The DNA was then digested with restriction enzymes (BfaI, CviAII, MseI and NdeI) and subsequently ligated at the proximal end of telomeres and DNA fragments with doubled-stranded TeSLA adapters with specific primer sequence for PCR. Multiple PCR reactions were performed, and their products were resolved on a 0.85% agarose gel (1.5 V/cm for 19 h). Southern blotting was used to quantify amplified telomeres. TeSLA raw data consist of a series of band sizes for each sample that are then used to calculate mean TL and proportions of short telomeres. For this study, we focus on the proportion of telomeres shorter than 3 kilobase (kb) (TeSLA3kb) because these telomeres are not typically captured by SB. Each sample was measured once. Notably, on average, the mean hematopoietic TL, measured by TeSLA is about 3 kb shorter than that measured by SB. This difference reflects the ability of TeSLA to detect telomeres < 3 kb and the use of different restriction enzymes in generating the terminal restriction fragments.

Monoplex qPCR TL assay was modified from original method as described (18, 21). Briefly, the qPCR assay quantifies the relative amount of telomeric DNA, expressed as the ratio of telomeric PCR product to that of a single copy gene (T/S). Primers used for the telomeric assay were Telo FP [5’-CGGTTT(GTTTGG)5GTT-3’] and Telo_RP [5’-GGCTTG(CCTTAC)5CCT-3’]2 and for the single-copy gene (36B4) assay were 36B4_FP [5’-CAGCAAGTGGGAAGGTGTAATCC-3’] and 36B4_RP [5’-CCCATTCTATCATCAACGGGTACAA-3’]. Each sample was measured in triplicate and T/S ratio was calculated using the mean values. qPCR measurements were available for 73 out of the 75 donor-recipient pairs.

The precision of the three methods was assessed by calculating the intraclass correlation coefficient (ICC) between runs performed on different days in different sample sets: SB (N=50), TeSLA (N=10), and qPCR (N=50) with the following results: 0.98, 0.90, and 0.83, respectively.

### Statistical analysis

We used paired t-test for hematopoietic cell TL comparisons of donor pre-, and recipient post-HCT, and Pearson’s correlation coefficient and linear regression models for association analyses. The donor-recipient hematopoietic cell TL correlation analyses were also performed separately by donor type (related and unrelated).

The post-HCT change in all hematopoietic cell TL parameter was calculated as the difference between the parameter in donors pre-HCT and in recipients three months post-HCT. Mean hematopoietic cell TL, measured by SB (SBmTL) or TeSLA (TeSLAmTL), is presented in kb; proportion of TeSLA3kb is presented in percent (%). Mean hematopoietic cell TL, measured by qPCR (qPCRmTL), is presented in T/S units. For assessing ‘regression to the mean’ (RTM) phenomenon in the relationship between donor hematopoietic cell TL and its magnitude of shortening, we calculated the corrected correlation coefficient (22). We used linear regression models for multivariate analyses. We set p<0.01 for statistical significance to minimize the effect of multiple testing.

The research use of blood samples and clinical information was approved by the National Marrow Donor Program IRB. All study participants provided written informed consent for participation in the BMT-CTN 1202 protocol (NCT01879072) and the CIBMTR repository and database protocols (NCT00495300, and NCT01166009, respectively).

## Results

### Characteristics of recipients and donors

Demographic characteristics of recipients and donors and key transplant factors are summarized in **Table 1**. The recipients (median age = 55 years; range = 3-74 years) were mainly white (86%) and 60% were males. The majority (66.7%) received HCT for a malignant disease, from unrelated donors (60%), and peripheral blood stem cell source (80%). The donors were 51% males. The unrelated donors had a median age of 30.5 years (range = 19.7-51.6 years), while the related donors had a median age of 48.4 years (range = 14.7-73.7 years). The recipients’ age correlated with the donors’ age for related recipient-donor pairs (r=0.83 in all related, and r=0.92 in matched siblings; p<0.0001), but not for unrelated pairs (r=0.06; p=0.71) (**Supplemental Figure S2**).

**Table 1:** Characteristics of patient and HCT-related factors.

Variables	N (%)/Median (range)
**Recipients’ age at HCT (years)**	55 (3-74)
**Donors’ age**	34 (15-74)
**Donors’ sex**	
* Male*	38 (50.7)
* Female*	37 (49.3)
**Recipients’ sex**	
* Male*	45 (60)
* Female*	30 (40)
**Recipients’ race**	
* White*	64 (85.3)
* Other*	11 (14.7)
**Disease**	
* Malignant (Leukemia or lymphoma)*	50 (66.7)
* MDS or MPN*	19 (25.3)
* Non-malignant*	6 (8.0)
**Donors’ type**	
* Matched sibling*	25 (33.3)
* Haploidentical*	5 (6.7)
* Unrelated donor*	45 (60.0)
**Stem cell source**	
* Bone marrow*	15 (20)
* Peripheral blood*	60 (80)
**Conditioning regimen**	
* Myeloablative*	48 (64.0)
* Nonmyeloablative*	7 (9.3)
* Reduced intensity*	20 (26.7)

### The relation between age and hematopoietic cell TL parameters of donors pre-HCT and recipients three months post-HCT

**Figure 1:**
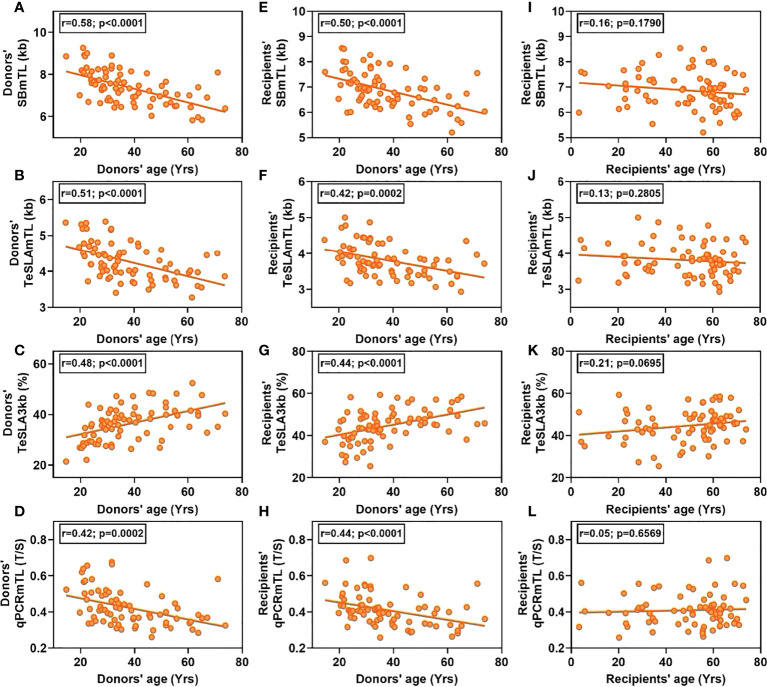
Correlations of hematopoietic cell TL parameters vs. age in donors and recipients. Left column **(A–D)**, donors’ hematopoietic cell TL parameters vs. donors’ age. Middle column **(E–H)**, recipients’ hematopoietic cell TL parameters vs. donors’ age. Right column **(I–L)**, recipients’ hematopoietic cell TL parameters vs. recipients’ age.

Both donors’ pre-HCT and recipient’s post-HCT hematopoietic cell TL parameters showed vast inter-individual variations (**Table 2**). Donors’ hematopoietic cell TL parameters correlated with donors’ age regardless of the TL measurement method (**Figures 1A–D**). The recipients’ hematopoietic cell TL parameters post-HCT also correlated with the donors’ age (**Figures 1E–H**) but not the recipients’ own age (**Figures 1I–L**).

**Table 2:** Hematopoietic cell TL parameters of donors pre-HCT and recipients post-HCT.

Assay	N	Donors	Recipients
		Mean (SD)	
SBmTL (kb)	75	7.4 (0.8)	6.8 (0.7)
TeSLAmTL (kb)	75	4.3 (0.5)	3.8 (0.4)
qPCRmTL (T/S)	73	0.42 (0.1)	0.41 (0.1)
TeSLA3kb (%)	75	36.2 (6.7)	44.6 (7.7)

SD, standard deviation; Kb, kilobase.

### Hematopoietic cell TL dynamics during the first three months post HCT

**Figure 2:**
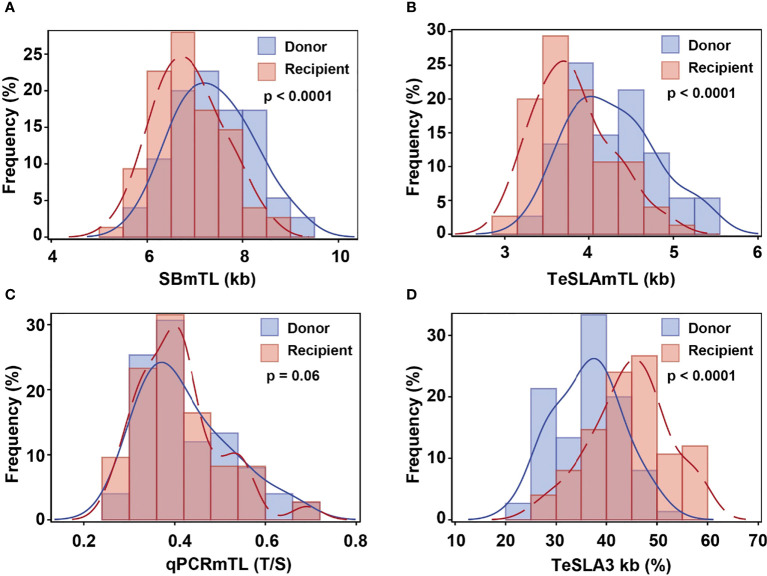
Hematopoietic cell TL distributions in donors pre-HCT and recipients three months post-HCT by assay [**(A)** SB; **(B)** qPCR; **(C, D)** TeSLA]. Blue line denotes donors’ pre-transplant hematopoietic cell TL and red line denotes recipients’ post-transplant hematopoietic cell TL.

SB and TeSLA detected substantial but highly variable shortening of telomeres in donated hematopoietic cells. This amounted to 0.52 ± 0.34 kb (mean ± SD) for SBmTL (p<0.0001), and 0.47 ± 0.34 kb for TeSLAmTL (p<0.0001) (**Figures 2A, B**). In contrast, qPCR detected little, if any, post-HCT shortening compared with that of the donors, qPCRmTL = 0.014 ± 0.06 T/S (p=0.06) (**Figure 2C**). TeSLA also detected an 8.3 ± 5.9% increase in the frequency of hematopoietic cell telomeres <3 kb in the recipients compared with the donors pre-HCT (p<0.0001) (**Figure 2D**).

**Figure 3:**
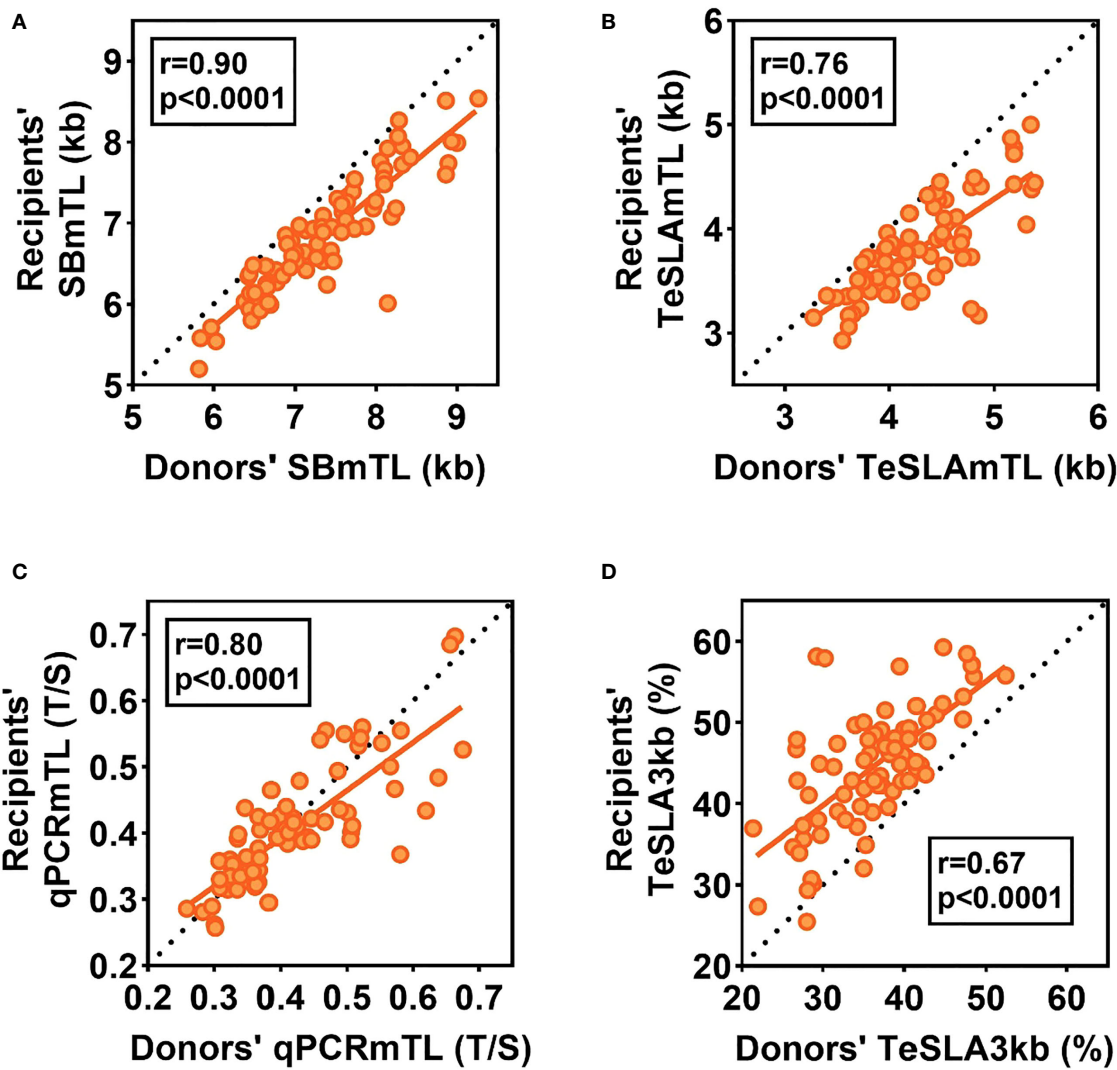
Relations of hematopoietic cell TL parameters between donors pre-HCT and recipients three months post-HCT. The dotted lines are the lines of identity. Data below the the line of identity for **(A-C)** denote shorter hematopoietic cell TL in recipients post-HCT than in donors pre-HCT. Data above the line of identity denote longer hematopoitic cell TL in recipients post-HCT than in donors’ pre-HCT. For **(D)**, data above the line of identity denotes a higher proportion of telomeres < 3kb in hematopoietic cells of recipients post-HCT than in donors pre-HCT, while data below the line of identity indicate a lower proportion of telomeres< 3kb post-HCT.

Based on SBmTL and TeSLAmTL, hematopoietic cell TL was shorter in all recipients than in donors (**Figures 3A, B**). In contrast, based on qPCRmTL, hematopoietic cell TL was shorter in only 42/73 recipients (57.5%) (**Figure 3C**). Based on TeSLA3kb, 72/75 (96%) of the recipients showed a higher proportion of telomeres < 3kb than the donors (**Figure 3D**).

The changes in the hematopoietic cell TL parameters during the three-month post HCT significantly correlated with the donors’ pre-transplant hematopoietic TL parameters (r= -0.41 for SBmTL; -0.51 for TeSLAmTL; and -0.45 for qPCRmTL; p<0.001 for all; and r= 0.27, for TeSLA3kb; p=0.02). However, these correlations became non-significant after correcting for regression to the mean (corrected r = -0.19, p = 0.10 for SBmTL, r = -0.15, p = 0.20 for TeSLAmTL, r = -0.13, p = 0.26 for qPCRmTL, and r = 0.16, p= 0.17 for TeSLA3kb).

### The relationships of hematopoietic cell TL parameters between recipients and donors

The recipients’ post-HCT hematopoietic cell TL parameters were strongly correlated with the donors’ hematopoietic cell TL parameters pre-HCT for all measurement methods (r= 0.90 for SBmTL; 0.76 for TeSLAmTL, 0.80 for qPCRmTL, and 0.67 for TeSLA3kb; all p<0.0001) (**Figures 3A–D**). Further details of the associations of the recipients’ hematopoietic cell TL parameters with donors’ age, other demographics, and transplant-related factors, are summarized in **Supplemental Table S1**.

Multivariate analyses that defined the recipients’ hematopoietic cell TL parameters post-HCT as the dependent variables, and donors’ age and hematopoietic cell TL parameters pre-HCT as the independent parameters, indicated that donors’ hematopoietic cell TL parameters respectively explained 81%, 56%, 65% and 44% of the variations in recipients’ SBmTL, TeSLAmTL, qPCRmTL and TeSLA3kb (p<0.0001 for all methods). This relationship was independent on donors’ age (p>0.3 for all methods) (**Supplemental Table S2**).

## Discussion

Findings of this work indicate that a highly variable and massive TL shortening of the donors’ hematopoietic cells (equivalent to ~0.50 TL shortening with 0.35 SD by SBmTL or TeSLAmTL) occurs during the first three months post-HCT as cells regenerate the recipients’ hematopoietic system. Despite variations, the donors’ hematopoietic cell TL pre-HCT was the principal determinant of the recipients’ hematopoietic cell TL post-HCT. Moreover, and on average, older donors have shorter hematopoietic cell TL than younger ones. Recipients of HCT from older donors are thus more likely to have shorter hematopoietic cell telomeres than recipients of HCT from younger donors. Our findings are relevant for HCT recipients for two major reasons: (i) the donors’ hematopoietic cell TL sets the recipients’ hematopoietic cell TL trajectories for their remaining lives, and (ii) recent studies infer that hematopoietic cell TL plays a causal role in aging-related diseases that also affect long-term survivors of HCT, including atherosclerotic cardiovascular disease (CVD) and certain cancers (23, 24).

In the general population, hematopoietic cell TL tracks with age, meaning that individuals maintain their relative TL ranking throughout their adult life (25). The massive shortening of hematopoietic cell TL early during the post-HCT means that the recipients’ hematopoietic cell TL is typically shorter than that of the donors for decades (26), if not their remaining life course. Donated hematopoietic cells with short telomeres due to older donors’ age or other factors might thus contribute to the increased CVD risk in long-term survivors of HCT [reviewed in (27)]. This TL-mediated CVD risk might be amplified because short telomeres in donated hematopoietic cells experience further shortening post-HCT. The role TL may play in subsequent cancer risk after HCT may be confounded by the complicated course of relapse of hematologic malignancy, and the development of new cancers associated with radiation regimens [reviewed in (28)]. Hematopoietic cell TL may, nonetheless, play a role in cancer survival post-HCT, as shown for several cancers in the general population (29).

Our study also underscores methodological matters with ramifications to design and findings of studies that examine post-transplant hematopoietic cell TL dynamics. First, as the TeSLA results match the SB results, TeSLA might provide a unique opportunity to examine the role shortest telomeres, which signal cessation of cell replication (30, 31), relevant to HCT outcomes. Second, as shown by their relatively lower ICC, qPCR measurements are known to be less precise than those generated by SB (32, 33). Still, the method was sufficiently reliable in capturing cross-sectional variation in hematopoietic cell TL in both donors and recipients, but showed suboptimal ability to capture hematopoietic cell TL shortening post-HCT. Based on the qPCR results, about 40% hematopoietic cells showed longer telomeres post-HCT than those of the corresponding donor pre-transplant. TL ‘elongation’ after HCT was also observed in a previous study of 124 nonmyeloablative allo-HCT patients, where qPCR TL change measured 9-15 months after HCT ranged between -50% to +29% of that of the donor (16). The effect of imprecise TL measurements in cross-sectional vs. longitudinal studies has been previously shown in the general population (33, 34). Third, the biological meaning of the T/S qPCR output provides minimal clinical insights, particularly in the absence of interlaboratory standardization and assay sensitivity to pre-analytic factors (35). In contrast, when express in TL units, the biological meaning of 0.5 kb shortening, for instance, is clear and may be relevant in assessing comorbidity risks in HCT survivors; for example, hematopoietic cell TL is shorter by about 0.3 kb in patients with CVD compared with their peers (36, 37). Relatedly, previous studies highlighted the suboptimal performance of qPCR TL assay in clinical setting particularly for patients with telomere-biology disorders who have very short TL (38, 39).

Finally, we acknowledge that our study is under-powered to evaluate associations of hematopoietic cell TL parameters pre-and post- HCT with patient outcomes.

In conclusion, we have characterized hematopoietic cell TL dynamics during the first three months post-HCT. We observed a massive but highly variable telomere shortening and a buildup of short telomeres during this time. We also showed the uncoupling in HCT recipients of hematopoietic cell TL from their own chronological age. This finding might be of interest not only to hematologists but also gerontologists. A long-term monitoring in HCT recipients of hematopoietic cell TL dynamics is essential for understanding the role of telomeres in the long-term survival post HCT and aging in general.

## Data availability statement

Deidentified data from this study are available upon request from the corresponding author (Shahinaz Gadalla; email: gadallas@mail.nih.ogv). Data access permission will require a material transfer agreement

## Ethics statement

The research use of blood samples and clinical Information was approved by the National Marrow Donor Program IRB. All study participants provided written informed consent for participation in the BMT-CTN 1202 protocol (NCT01879072) and the CIBMTR repository and database protocols (NCT00495300, and NCT01166009, respectively).

## Author contribution

Designed research: AA, SG. DNA extraction and Telomere length measurement: TL, CD, AHu, AA. Facilitated data and sample access: SS, AHo, JL, WS. Statistical analysis: SV, HK, SG. Drafted Manuscript: TL, SG, AA. Critical Review: All co-authors. All authors contributed to the article and approved the submitted version.

## Funding

The study is funded by the NIH grant U01AG066529, and by the intramural program of the National Cancer Institute, NIH. The Cancer Genomics Research Laboratory is funded with federal funds from the NCI, NIH, under NCI Contract 75N910D00024. T-PL is supported by NSF grant 2032119, NIH grants 1U01AG066529, 3U01AG066529-02S1, NCI contract 75N91019P00829, and New Jersey Alliance for Clinical and Translational Science Career Development Award NJACTS KL2 TR003018. The CIBMTR is supported primarily by Public Health Service U24CA076518 from the NCI, the National Heart, Lung and Blood Institute and the National Institute of Allergy and Infectious Diseases; HHSH250201700006C from the Health Resources and Services Administration; and N00014-21-1-2954 and N00014-20-1-2832 from the Office of Naval Research. Support for this study was provided by grants U10HL069294 and U24HL138660 to the Blood and Marrow Transplant Clinical Trials Network (BMT-CTN) from the National Heart, Lung, and Blood Institute and the National Cancer Institute. The content is solely the responsibility of the authors and does not necessarily represent the official views of the NIH and the above-mentioned parties

## Conflict of interest

Author’s CD and AH were employed by Leidos Biomedical Research, Inc.

The remaining authors declare that the research was conducted in the absence of any commercial or financial relationships that could be construed as a potential conflict of interest.

## Publisher’s note

All claims expressed in this article are solely those of the authors and do not necessarily represent those of their affiliated organizations, or those of the publisher, the editors and the reviewers. Any product that may be evaluated in this article, or claim that may be made by its manufacturer, is not guaranteed or endorsed by the publisher.

## Supplementary material

Supplementary Figure 1 Summarizes the correlations between mean (m) hematopoietic cell TL by the three measurement methods.
