# Supplementary material for: Decoupling blood telomere length from age in recipients of allogeneic hematopoietic cell transplant in the BMT-CTN 1202
Source: Front Immunol. 2022 Oct 3;13:966301. doi: 10.3389/fimmu.2022.966301 (PMC9574912; doi:10.3389/fimmu.2022.966301)

| **Table S1: Univariable associations of recipients’ HCTL three months after HCT with potential demographics and clinical factors** | | | | | | | | |
| --- | --- | --- | --- | --- | --- | --- | --- | --- |
| Variables | Recipient post-HCT | | | | | | | |
|  | SBmTL (Kb) | | TeSLAmTL (kb) | | qPCRmTL (logT/S) | | TeSLA<3kb (%) | |
|  | β | ***P*** | β | ***P*** | β | ***P*** | β | ***P*** |
| Donor TL | 0.82 | <0.0001 | 0.66 | <0.0001 | 0.76 | <0.0001 | 0.76 | <0.0001 |
| Donor age | -0.02 | <0.0001 | -0.01 | 0.0002 | -0.006 | 0.0004 | 0.24 | <0.0001 |
| Recipient Age | -0.006 | 0.18 | -0.003 | 0.28 | 0.001 | 0.65 | 0.09 | 0.07 |
| Donor Sex  (Male vs. female) | -0.03 | 0.86 | -0.0001 | 0.99 | 0.05 | 0.35 | -0.59 | 0.74 |
| Recipient sex  (Male vs. female) | 0.15 | 0.38 | 0.19 | 0.07 | 0.04 | 0.37 | -2.38 | 0.19 |
| Recipient-donor sex mismatch | 0.21 | 0.22 | 0.10 | 0.35 | 0.06 | 0.27 | -0.10 | 0.95 |
| Disease  (malignant vs. benign) | 0.61 | 0.05 | 0.18 | 0.34 | 0.18 | 0.09 | -1.16 | 0.72 |
| Conditioning intensity  (MAC vs. RIC) | 0.25 | 0.15 | 0.06 | 0.52 | -0.05 | 0.34 | -1.76 | 0.34 |
| TBI conditioning  (Yes vs. No) | 0.01 | 0.94 | -0.06 | 0.59 | -0.006 | 0.90 | 0.05 | 0.98 |
| Donor type  (Related vs. unrelated) | -0.34 | 0.04 | -0.17 | 0.09 | -0.08 | 0.11 | 3.07 | 0.09 |
| Stem cell source  (Peripheral blood vs. Bone marrow) | 0.21 | 0.19 | 0.06 | 0.64 | 0.14 | 0.03 | -0.15 | 0.95 |
| Acute GVHD 2-4  (Yes vs. No) | 0.34 | 0.04 | 0.06 | 0.58 | 0.004 | 0.93 | -0.46 | 0.79 |

@ΔHCTL is calculated as (recipient post-HCT TL - donor pre-HCT TL).

| **Table S2: Multivariate regression model of recipients’ post hematopoietic cell TL parameters with the donor’s age and hematopoietic cell TL parameters pre-HCT** | | | | | | | | |
| --- | --- | --- | --- | --- | --- | --- | --- | --- |
| **Variables** | **Recipients’ mean hematopoietic cell TL** | | | | | | **Recipients’ TeSLA3kb (%)** | |
|  | **SBmTL (Kb)** | | **TeSLAmTL (kb)** | | **qPCRmTL^*^ (SD)** | |  |  |
| **Full Cohort** | | | | | | | | |
|  | **β** | **p** | **β** | **p** | **β** | **p** | **β** | **p** |
| Donors’ TL parameter^#^ | 0.84 | <0.0001 | 0.64 | <0.0001 | 0.73 | <0.0001 | 0.64 | <0.0001 |
| Donors’ age | 0.002 | 0.60 | -0.001 | 0.58 | -0.000 | 0.39 | -0.001 | 0.58 |
| Model R^2^ | 0.81 | | 0.56 | | 0.65 | | 0.44 | |
| ^*^On the log scale  ^#^Assay-specific mean hematopoietic cell TL or TeSLA3kb | | | | | | | | |

**Figure S1: Correlations between hematopoietic cell telomere length (HCTL) parameters by measurement methods**.

Upper panel (a-c) are from donor pre-transplant HCTL measurements. Lower panel (d-f) are from recipient post-transplant HCTL measurements


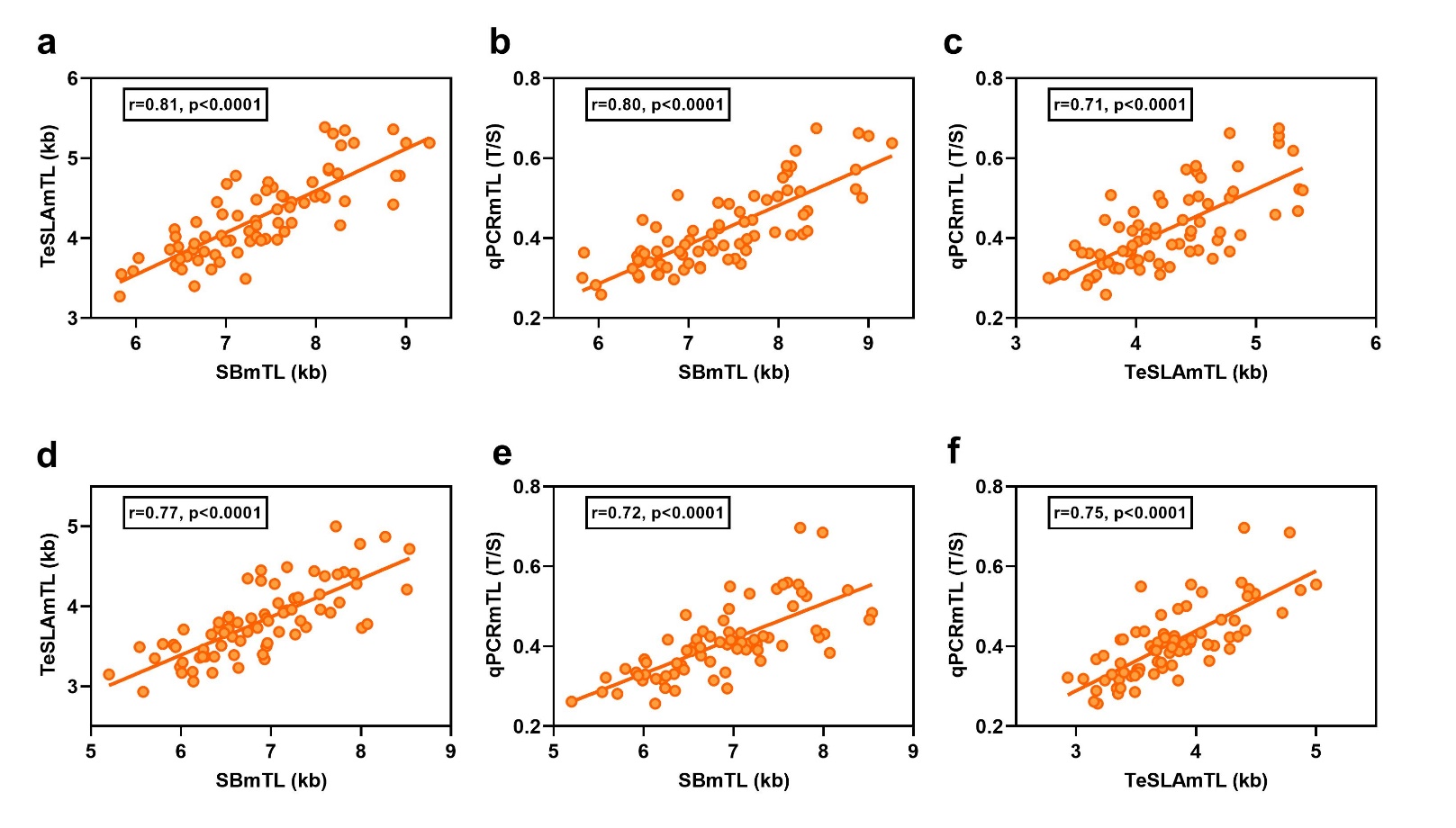


**Figure S2: The correlation between age of the recipients and donors by HCT donor type**.

Related donor HCTs are presented in blue, and unrelated donor HCTs are in red


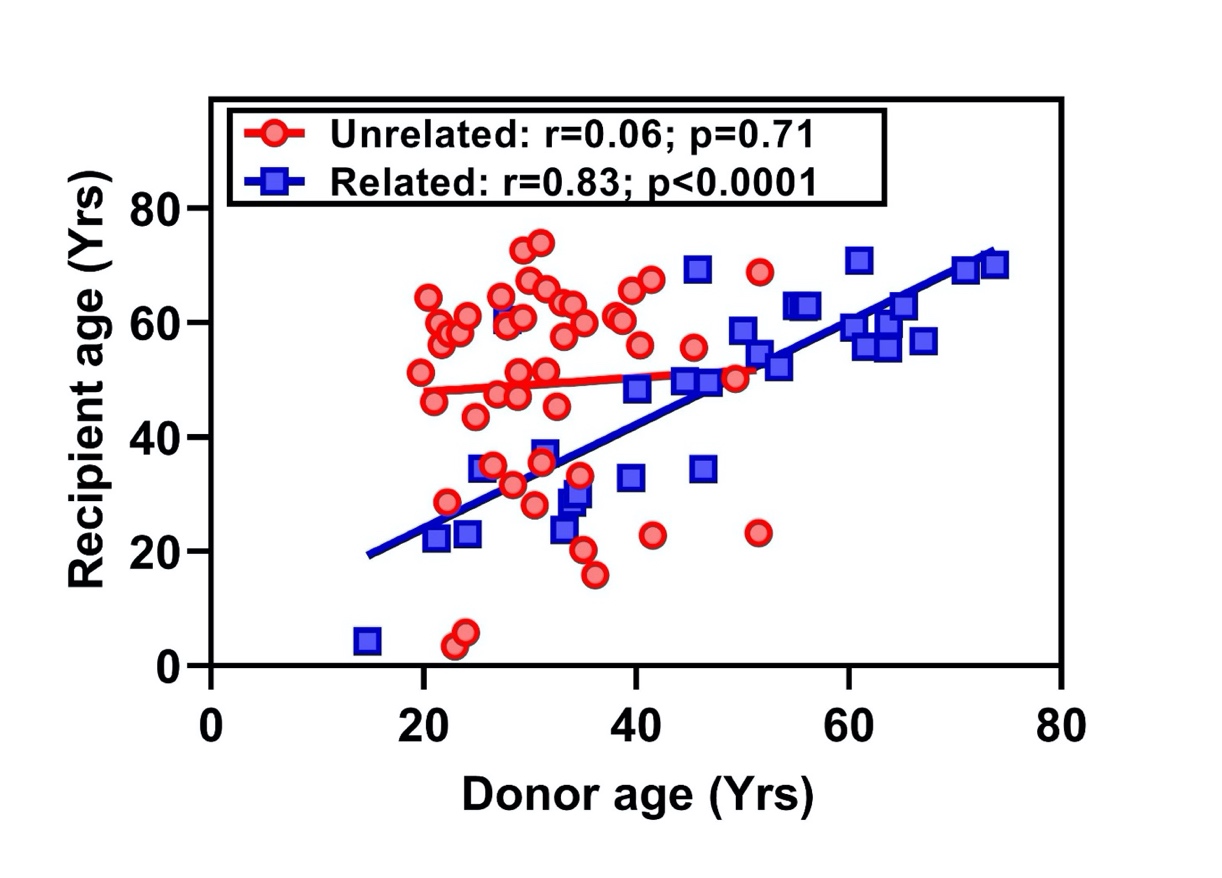

Supplement: Supplementary Figure 1 — Summarizes the correlations between mean (m) hematopoietic cell TL by the three measurement methods. [file DataSheet_1.docx]
